# Wogonin Improves Histological and Functional Outcomes, and Reduces Activation of TLR4/NF-κB Signaling after Experimental Traumatic Brain Injury

**DOI:** 10.1371/journal.pone.0030294

**Published:** 2012-01-17

**Authors:** Chien-Cheng Chen, Tai-Ho Hung, Yen-Ho Wang, Chii-Wann Lin, Pei-Yi Wang, Chun-Yen Lee, Szu-Fu Chen

**Affiliations:** 1 Department of Physical Medicine and Rehabilitation, Cheng Hsin General Hospital, Taipei, Taiwan, Republic of China; 2 Institute of Biomedical Engineering, National Taiwan University Taipei, Taiwan, Republic of China; 3 Department of Obstetrics and Gynecology, Chang Gung Memorial Hospital at Taipei and College of Medicine, Chang Gung University, Taipei, Taiwan, Republic of China; 4 Department of Physical Medicine and Rehabilitation, National Taiwan University Hospital and National Taiwan University College of Medicine, Taipei, Taiwan, Republic of China; 5 Departments of Physiology and Biophysics, National Defense Medical Center, Taipei, Taiwan, Republic of China; Universidad de Castilla-La Mancha, Spain

## Abstract

**Background:**

Traumatic brain injury (TBI) initiates a neuroinflammatory cascade that contributes to neuronal damage and behavioral impairment. This study was undertaken to investigate the effects of wogonin, a flavonoid with potent anti-inflammatory properties, on functional and histological outcomes, brain edema, and toll-like receptor 4 (TLR4)- and nuclear factor kappa B (NF-κB)-related signaling pathways in mice following TBI.

**Methodology/Principal Findings:**

Mice subjected to controlled cortical impact injury were injected with wogonin (20, 40, or 50 mg·kg^−1^) or vehicle 10 min after injury. Behavioral studies, histology analysis, and measurement of blood-brain barrier (BBB) permeability and brain water content were carried out to assess the effects of wogonin. Levels of TLR4/NF-κB-related inflammatory mediators were also examined. Treatment with 40 mg·kg^−1^ wogonin significantly improved functional recovery and reduced contusion volumes up to post-injury day 28. Wogonin also significantly reduced neuronal death, BBB permeability, and brain edema beginning at day 1. These changes were associated with a marked reduction in leukocyte infiltration, microglial activation, TLR4 expression, NF-κB translocation to nucleus and its DNA binding activity, matrix metalloproteinase-9 activity, and expression of inflammatory mediators, including interleukin-1β, interleukin-6, macrophage inflammatory protein-2, and cyclooxygenase-2.

**Conclusions/Significance:**

Our results show that post-injury wogonin treatment improved long-term functional and histological outcomes, reduced brain edema, and attenuated the TLR4/NF-κB-mediated inflammatory response in mouse TBI. The neuroprotective effects of wogonin may be related to modulation of the TLR4/NF-κB signaling pathway.

## Introduction

Traumatic brain injury (TBI) induces a complex series of inflammatory responses that contribute to neuronal damage and behavioral impairment [1]. Toll-like receptors (TLRs) are a family of signal transduction molecules known to activate the innate immune response following systemic bacterial infection and cerebral injury [2]. Among the TLRs, TLR4 has been shown to play an important role in initiating the inflammatory response in the damaged brain. Several animal studies have shown that both TLR4 mRNA and protein are upregulated following TBI [3]–[5]. TLR4-mediated signaling pathways mainly stimulate the activation of nuclear factor kappa B (NF-κB). This important nuclear transcription factor regulates many pro-inflammatory genes, e.g., cytokines, chemokines, cyclooxygenase-2 (COX-2), and matrix metalloproteinase-9 (MMP-9), mediators involved in the pathogenesis of TBI [6]. TLR4-deficient mice exhibited reduced infarct size and improved neurological recovery as well as less inflammatory response following cerebral ischemia [7], [8]. Furthermore, neurons from TLR4 mutant mice were protected against energy deprivation-induced cell death, which was associated with decreased activation of pro-apoptotic c-Jun N-terminal kinase signaling [7]. These studies suggest that pharmacological inhibition of TLR4/NF-κB signaling may be a useful strategy for protection of the injured brain.

Wogonin, 5,7-dihydroxy-8-methoxyflavone, is one of the major flavonoids found in the root of the Chinese herb *Scutellaria baicalensis* Georgi (also called Huang-Qin), which is widely used in treating allergic and inflammatory diseases [9]. Wogonin has been shown to exert potent anti-inflammatory effects in both *in vitro* and *in vivo* studies. For example, it has been demonstrated that wogonin suppresses lipopolysaccharide (LPS)-induced production of nitric oxide (NO), prostaglandin E2, and pro-inflammatory cytokines in immune cells such as macrophages and microglial cells [10]–[12] and reduces migration in microglial cells via inhibition of NF-κB activity [13]. In addition, treatment with wogonin was found to alleviate inflammatory responses caused by skin inflammation and carrageenan-induced hindpaw edema in animal studies [14], [15]. Increasing evidence suggests that wogonin may have neuroprotective effects in the injured brain. Wogonin attenuated the death of hippocampal neurons and inhibited microglia activation in global ischemia and excitotoxic injury models [11]. Furthermore, wogonin also reduced early ischemic brain injury and improved acute behavioral dysfunctions caused by focal cerebral ischemia [16], [17]. In addition, wogonin attenuated excitotoxic and oxidative stress-induced neuronal damage in primary cultured rat cortical cells [18] and reduced neuronal damage caused by exposure to oxygen and glucose deprivation in cultured rat hippocampal slices [19]. Despite evidence indicating the benefits of wogonin treatment to early neurological recovery in stroke models, there is a lack of data describing the long-term effects of wogonin on functional recovery or cell survival in the injured brain. In particular, the neuroprotective effects of wogonin on TBI have not been established.

The aim of the present study was to investigate the protective effects of wogonin on neuronal damage, brain edema, and functional impairment after TBI. We further examined whether wogonin could attenuate TBI-induced activation of the TLR4/NF-κB signaling pathway in the pericontusional area.

## Results

### Metabolic characteristics

There were no significant differences in the levels of plasma blood urea nitrogen (BUN), creatinine (CRE), alanine aminotransferase (ALT), or aspartate aminotransferase (AST) between the vehicle-treated and 40 mg·kg^−1^ wogonin-treated injured mice (Table 1). The mice in both groups lost a small proportion of body weight (∼6%) during the initial 2 days after CCI, but regained baseline weight within 7 days. No significant difference in body weight was detected between groups treated with wogonin or vehicle (*P* = 0.8261, data not shown).

**Table 1:** Metabolic characteristics of the sham control, mice treated with DMSO and 40 mg·kg^−1^ wogonin.

		Day 1		Day 28	
	Sham	Vehicle	Wogonin	Vehicle	Wogonin
BUN (mg/dL)	20.00±1.30	21.20±1.50	19.30±1.60	20.50±1.20	18.50±1.80
CRE (mg/dL)	0.27±0.07	0.20±0.10	0.27±0.07	0.27±0.09	0.33±0.07
ALT (mg/dL)	28.30±10.20	27.20±2.60	25.80±2.10	22.90±2.30	23.70±2.10
AST (mg/dL)	46.70±8.50	46.00±9.80	35.70±6.30	43.00±8.20	45.70±9.20

Values are expressed as means ± SEM. n = 7/groups. BUN: blood urea nitrogen; CRE: creatinine; AST: aspartate aminotransferase; ALT: alanine aminotransferase.

### Post-injury wogonin administration improves neurobehavioral recovery following TBI Rotarod test

**Figure 1:**
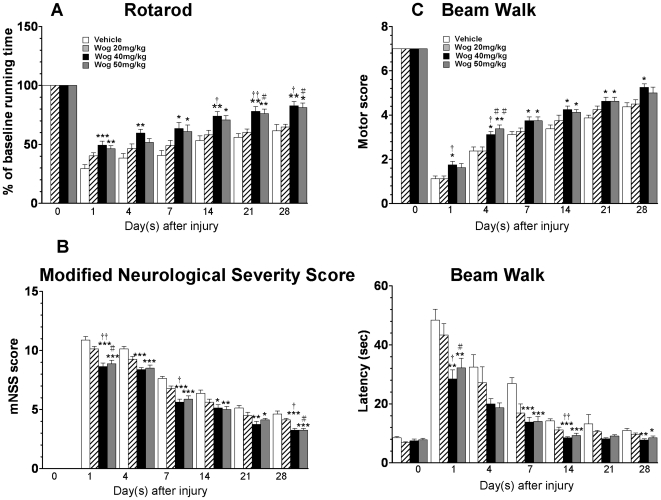
Effects of 3 different doses of wogonin on functional outcomes in contusion-injured mice. (**A**) Compared to the vehicle treatment, 20 mg·kg^−1^ wogonin treatment did not significantly alter rotarod performance. On days 1–28, 40 mg·kg^−1^ wogonin-treated mice performed significantly better than the vehicle-treated mice. On days 1, 7, 14, 21, and 28, the 50 mg·kg^−1^ wogonin-treated mice had better rotarod performance than the vehicle-treated mice. No significant differences were observed between the 40 mg·kg^−1^ and 50 mg·kg^−1^ wogonin treatment groups at any time point. (**B**) No difference in modified Neurological Severity Score (mNSS) was detected between the 20 mg·kg^−1^ wogonin and vehicle treatment groups. On days 1–28, the mNSSs were significantly lower in the 40 mg·kg^−1^ and 50 mg·kg^−1^ dose groups than in the vehicle-treated group. No significant differences were observed between the 40 mg·kg^−1^ and 50 mg·kg^−1^ treatment groups. (**C**) During the beam walk test, no significant differences were observed between the 20 mg·kg^−1^ wogonin-treated and vehicle-treated groups. Significant differences were observed in the hindlimb motor scores between the 40 mg·kg^−1^ wogonin-treated and vehicle-treated groups on post-injury test days 1–28 and between the 50 mg·kg^−1^ dose group and vehicle group on days 4, 7, 14, and 21. Differences in hindlimb motor scores between the 40 mg·kg^−1^ and 50 mg·kg^−1^ groups were not significant. Beam walk latencies were significantly shorter for both the 40 mg·kg^−1^ and 50 mg·kg^−1^ groups than for the vehicle group, on days 1, 7, 14, and 28; however, no significant difference was observed between the 2 wogonin-treated groups. Values are presented as mean ± SEM; *P<0.05, **P<0.01, ***P<0.001 versus vehicle-treated injured mice. ^†^ *P*<0.05, ^††^ *P*<0.01: 20 mg·kg^−1^ wogonin-treated mice versus the 40 mg·kg^−1^ wogonin-treated mice. ^#^ *P*<0.05: 20 mg·kg^−1^ wogonin-treated mice versus the 50 mg·kg^−1^ wogonin-treated mice (n = 8 mice/group at each time point).

We first conducted several sets of behavioral experiments to verify whether post-injury wogonin treatment could improve recovery from neurologic deficits. TBI induced persistent motor function impairment with a significant decrease in rotarod running time from 1 to 28 days in the vehicle-treated group (Fig. 1A). Treatment with 20 mg·kg^−1^ wogonin did not significantly alter rotarod performance compared with the vehicle-treated group. Nevertheless, performance on the rotarod test was significantly better for 40 mg·kg^−1^ wogonin-treated mice than for vehicle-treated mice on test days 1–28 after injury (all *P*<0.05). In addition, mice treated with 50 mg·kg^−1^ wogonin had better performance in the rotarod test at days 1, 7, 14, 21, and 28 than vehicle-treated mice (all *P*<0.05). There was no significant difference between 40 mg·kg^−1^- and 50 mg·kg^−1^-treated-groups at all tested time points.

### Modified neurological severity score

Injury in the left hemispheric cortex resulted in neurological deficits as measured by modified neurological severity score (mNSS) (Fig. 1B). There was no significant difference between 20 mg·kg^−1^ wogonin-treated and vehicle-treated groups at all tested time points. The mNSS scores were significantly lower in both the 40 mg·kg^−1^ and 50 mg·kg^−1^ wogonin-treated group than the corresponding vehicle-treated group on test days 1–28 after injury (all *P*<0.05). There was no significant difference between the 40 mg·kg^−1^- and 50 mg·kg^−1^-treated groups at all tested time points.

### Beam walk test

Marked impairment in beam walk performance including decrease in hindlimb motor scores and increase in beam walk latency (traversing latency) was observed on the first day after surgery, regardless of treatment (Fig. 1C). There was no significant difference between the 20 mg·kg^−1^ wogonin-treated and vehicle-treated groups at all tested time points. The decrease in hindlimb motor scores was significantly different between the 40 mg·kg^−1^ wogonin-treated group and vehicle-treated group on test days 1–28 after injury (all *P*<0.05) and between the 50 mg·kg^−1^-dose group and vehicle group on days 4, 7, 14, 21, and 28 (all *P*<0.05). However, differences in hindlimb motor scores between the 40 mg·kg^−1^ and 50 mg·kg^−1^ groups were not significant on any testing day (all *P*>0.05). Likewise, beam walk latencies were significantly shorter for both the 40 mg·kg^−1^ and 50 mg·kg^−1^ groups than for the vehicle group on days 1, 4, and 7 (all *P*<0.05), though no significant differences between the 2 wogonin-treated groups were found (Fig. 1C). Furthermore, though beam walk latencies on days 14, 21, and 28 were shorter for both the 40 mg·kg^−1^ and 50 mg·kg^−1^ groups than for the vehicle group, these differences did not reach statistical significance.

### Post-injury wogonin administration reduces cortical contusion volume, blood-brain barrier permeability, and brain edema

**Figure 2:**
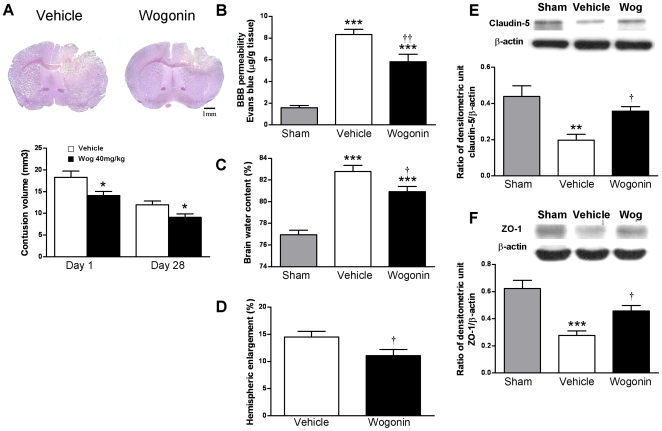
Effects of 40 mg·kg^−1^ wogonin treatment on cortical contusion volume, brain edema, and BBB permeability. (**A**) Representative cresyl violet-stained brain sections of vehicle- and 40 mg·kg^−1^ wogonin-treated mice 1 day post-TBI showing hypointense regions immediately below the impact site in the cortex. Scale bar is 1 mm. Quantification showed significantly smaller contusion volumes in wogonin-treated mice compared with vehicle-treated mice at days 1 and 28 post-TBI. (**B**) Wogonin-treated mice showed a significant decrease in the concentration of Evans blue (EB) in the ipsilateral hemisphere compared with vehicle-treated mice at day 1. (**C**) Brain water content in the ipsilateral hemisphere of 40 mg·kg^−1^ wogonin-treated mice was significantly lower than in vehicle-treated mice at day 1. (**D**) At day 1, hemispheric enlargement was significantly smaller in mice treated with 40 mg·kg^−1^ wogonin than in vehicle-treated mice. (**E, F**) Treatment with 40 mg·kg^−1^ wogonin also reversed TBI-mediated reduced expression of claudin-5 and zonula occludens 1 in traumatic cortical areas of the ipsilateral hemisphere at day 1 following TBI, as measured by western blot. Values are presented as mean ± SEM; **P<0.01, ***P<0.001 versus vehicle-treated injured mice. ^†^ *P*<0.05, ^††^ *P*<0.01 for the 40 mg·kg^−1^ wogonin-treated mice versus vehicle-treated mice. (n = 8 mice/group for cresyl violet staining and hemispheric enlargement, n = 7 mice/group for brain EB, brain water content, and western blot).

Since 40 mg·kg^−1^ wogonin treatment significantly improved neurological outcomes, we further investigated whether this treatment paradigm reduced neuronal damage, brain edema, and post-traumatic inflammation. Brain sections taken from controlled cortical impact (CCI) mice revealed a loss of cortical tissue in the ipsilateral parietal cortex as reflected by gross reductions in cresyl violet staining intensity (Fig. 2A). Quantification of cortical volume showed that at day 1 after CCI, there was a 22.9% reduction in contusion volume in 40 mg·kg^−1^ wogonin-treated mice compared with vehicle-treated controls. Contusion volume in the wogonin group was 14.1±1.0 mm^3^ versus 18.3±1.4 mm^3^ in the vehicle group (*P*<0.05; Fig. 2A). Long-term effects were evaluated at day 28 post-injury by which time the cortical contusion volume in the vehicle-treated TBI group was measured to be 12.0±0.9 mm^3^ (Fig. 2A). Treatment with wogonin resulted in reduction of lesion size by 24.2% to 9.1±0.8 mm^3^, which was significantly smaller than the vehicle-treated group (*P*<0.05; Fig. 2A).

Disruption of the blood-brain barrier (BBB) and brain edema can cause brain swelling and increased intracranial pressure following TBI, leading to secondary injury and cell death [20]. To assess whether the reduction in neurological deficits and brain injury after wogonin treatment correlated with reduced BBB disruption and brain edema, we next examined hemispheric enlargement and brain water content 1 day post-TBI. BBB permeability was determined by Evans blue (EB) dye extravasation. There was a marked increase in EB content in the ipsilateral hemisphere of the vehicle-treated TBI group as compared with the sham control (8.3±0.5 µg·g^−1^ versus 1.6±0.2 µg·g^−1^, *P*<0.001; Fig. 2B). TBI-induced increases in EB dye extravasation in the ipsilateral hemisphere were significantly reduced by wogonin treatment at day 1 post-TBI (8.3±0.5 µg·g^−1^ versus 5.8±0.7 µg·g^−1^, *P*<0.01). Because BBB breakdown may lead to accumulation of circulating fluid and contribute to brain edema [21], we further assessed whether wogonin treatment could reduce brain edema at day 1, when brain edema reached the maximum following CCI in mice. Brain water content, an indicator of brain edema, was significantly increased in the ipsilateral hemisphere by day 1 in vehicle-treated TBI mice compared with that of sham controls (82.8±0.6% versus 77.0±0.4%, *P*<0.001; Fig. 2C). Treatment with wogonin caused a reduction in the percent water content within the ipsilateral hemisphere cortex compared with the vehicle-treated group (80.9±0.5% versus 82.8±0.6%, *P*<0.05; Fig. 2C). Similarly, hemispheric enlargement was significantly smaller in wogonin-treated mice (10.9±1.1%) than in the vehicle-treated mice (14.5±1.1%, *P*<0.05; Fig. 2D).

We next sought to determine the effects of wogonin on 2 major proteins involved in tight junctions of the BBB, claudin-5 and zonula occludens (ZO)-1. TBI caused a significant decrease in both claudin-5 and ZO-1 expression at day 1 after injury (Figs. 2E & F). However, this apparent loss of claudin-5 and ZO-1 was reversed after wogonin treatment. Protein expression of claudin-5 and ZO-1 in the injured cortex of wogonin-treated mice was increased to 181.6% (*P*<0.05) and 164.8% (*P*<0.05) of the vehicle group, respectively.

### Post-injury wogonin administration reduces neuronal and apoptotic cell death

**Figure 3:**
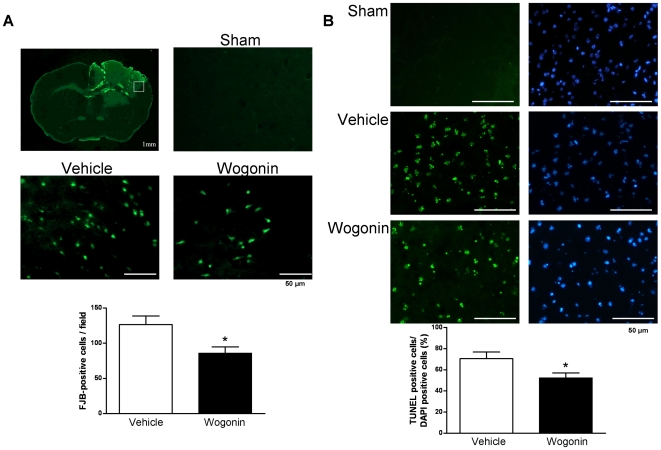
Effects of 40 mg·kg^−1^ wogonin treatment on neuronal degeneration and apoptotic cell death. (**A**) Brain atlas of coronal sections of a core contusional region at 0.74 mm from the bregma. Quantification analysis indicated that wogonin-treated mice had significantly fewer degenerating neurons than vehicle-treated mice in the cortical contusion margin at day 1 post-TBI. The total number of Fluoro-Jade B (FJB)-positive cells is expressed as the mean number per field of view (1.3 mm^2^). The scale bar is 50 µm. (**B**) Representative terminal deoxynucleotidyl transferase-mediated dUTP-biotin nick end labeling (TUNEL) staining (green)- and DAPI (blue)-stained brain sections of a sham-injured control, a wogonin-treated mouse, and a vehicle-treated mouse at day 1 post-TBI. Quantification showed that wogonin-treated mice had significantly fewer TUNEL-positive cells than the vehicle-treated mice in the cortical contusion margin at day 1 post-TBI. The percentage of TUNEL-positive cells is expressed as the number of TUNEL-stained nuclei/the total number of DAPI-stained nuclei. Sections were stained with DAPI (blue) to show all nuclei. The scale bar is 50 µm. Values are presented as means ± SEM; *P<0.05 versus vehicle-treated injured mice (n = 7 mice/group).

We used Fluoro-Jade B (FJB) and terminal deoxynucleotidyl transferase-mediated dUTP-biotin nick end labeling (TUNEL) staining to examine whether neuronal death was decreased in the pericontusion region of wogonin-treated mice. Since FJB and TUNEL reactivity has been shown to peak 1 day after CCI [22], [23], the day 1 time point was chosen. Both FJB-positive cells with neuronal morphology and TUNEL-positive cells were evident at day 1 after injury in the cortical contusion margin (Figs. 3A & B) and striatum in the ipsilateral but not the contralateral hemisphere. Wogonin-treated mice had significantly fewer FJB-positive neurons around the injured cortical areas at day 1 post-injury than observed in the vehicle-treated group (85.7±9.0 versus 126.7±12.1 cells/field, *P*<0.05; Fig. 3A). Furthermore, TUNEL staining showed that the number of apoptotic cells increased markedly in cerebral tissues surrounding the injured area in TBI groups at 24 h post-injury. Significantly fewer TUNEL-positive cells were found around injured cortical areas in wogonin-treated mice (52.3±4.7%) than in vehicle-treated controls (70.4±6.3%, *P*<0.05; Fig. 3B).

### Post-injury wogonin administration reduces neutrophil infiltration and microglial activation

**Figure 4:**
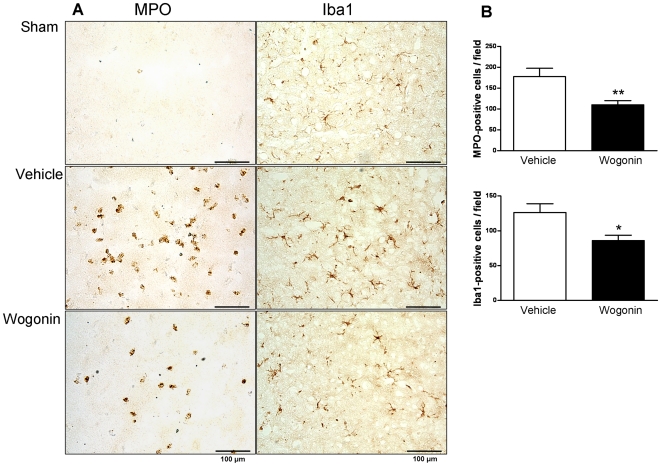
Effects of 40 mg·kg^−1^ wogonin treatment on neutrophil infiltration and microglial activation. (**A**) Representative myeloperoxidase (MPO)- and anti-ionized calcium binding adaptor molecule 1 (Iba1)-stained brain sections from a sham-injured control, a wogonin-treated, and a vehicle-treated mouse at day 1 post-TBI. (**B**) Cell count analysis indicated that wogonin-treated mice had significantly fewer infiltrating neutrophils and activated microglia/macrophages than vehicle-treated mice in the cortical contusion margin at day 1 post-TBI. The total number of MPO- and Iba1-positive cells is expressed as the mean number per field of view (1.3 mm^2^). The scale bar is 100 µm. Values are presented as means ± SEM; *P<0.05, **P<0.01 versus vehicle-treated TBI mice (n = 7 mice/group).

The above results suggest that wogonin may improve functional recovery and reduce neuronal damage following TBI. We further explored whether wogonin treatment directly influenced post-injury early inflammatory events. Since neutrophil infiltration and microglial activation may contribute to TBI-induced tissue injury, we examined whether wogonin treatment had any effects on infiltrated neutrophils and activated microglia/macrophages. We found that TBI produced a robust infiltration of neutrophils in the injured cortex, primarily in the contusion core and margin, in vehicle-treated mice. Furthermore, wogonin-treated mice had significantly fewer neutrophils in the pericontusion area than did vehicle-treated mice (110.0±9.9 versus 177.4±20.0 cells/field, *P*<0.05; Figs. 4A & B). With regard to the microglial response, there was a significant increase in ionized calcium binding adaptor molecule 1 (Iba1)-positive microglia within the injured cortex in the vehicle-treated group. This increase was attenuated by wogonin treatment as well (86.0±7.8 versus 126.3±12.6 cells/field, *P*<0.01; Fig. 4A & B).

### Post-injury wogonin administration reduces IL-1β, IL-6, MIP-2, and COX-2 expression, but has no effect on MCP-1 expression after TBI

**Figure 5:**
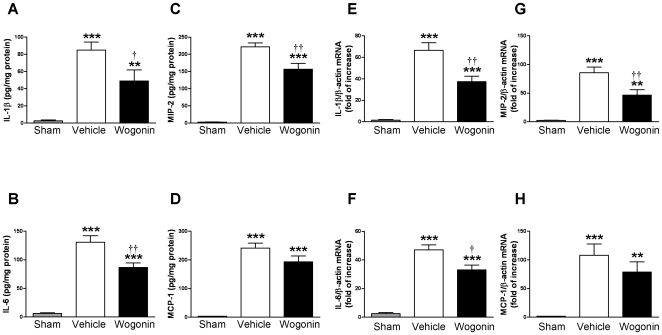
Effects of 40 mg·kg^−1^ wogonin treatment on protein and mRNA expression of cytokines and chemokines. (**A, B, C, D**) Bar graphs of interleukin (IL)-1β, IL-6, macrophage inflammatory protein (MIP)-2, and monocyte chemoattractant protein (MCP)-1 protein concentrations in the ipsilateral cortices of sham control, vehicle-treated, and 40 mg·kg^−1^ wogonin-treated mice at day 1 post-injury. Wogonin significantly attenuated injury-induced increases in IL-1β, IL-6, and MIP-2 protein concentrations, but had no effect on MCP-1 protein concentration compared with vehicle-treated TBI mice. (**E, F, G, H**) Bar graphs demonstrating IL-1β, IL-6, MIP-2, and MCP-1 mRNA expression in the ipsilateral cortices of sham control, vehicle-treated, and 40 mg·kg^−1^ wogonin-treated mice 6 h post-injury. Wogonin significantly inhibited injury-induced expression of IL-1β, IL-6, and MIP-2 mRNA in the ipsilateral cortices. There was no significant difference in MCP-1 mRNA transcript levels between the wogonin-treated and vehicle-treated groups of mice subjected to TBI. Values are presented as means ± SEM; **P<0.01, ***P<0.001 versus sham control, and ^†^ *P*<0.05, ^††^ *P*<0.01 for wogonin-treated mice versus vehicle-treated TBI mice (n = 7 mice/group).

**Figure 6:**
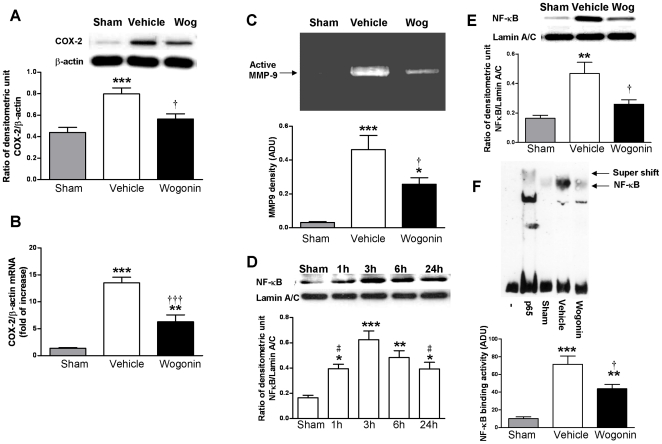
Effects of 40 mg·kg^−1^ wogonin on COX-2 expression, MMP-9 and NF-κB activation. (**A**) Representative immunoblots and densitometry analysis showed a significant decrease in COX-2 protein levels in the ipsilateral hemispheres of wogonin-treated injured mice at day 1 compared with vehicle-treated injured mice. (**B**) Bar graphs showed that wogonin significantly reduced COX-2 mRNA expression in the ipsilateral hemispheres compared with vehicle-treated injured mice at 6 h post-TBI. (**C**) Representative zymography and densitometry analysis showed that MMP-9 activity was significantly decreased in wogonin-treated mice compared with vehicle-treated mice at day 1. (**D**) Representative immunoblots of nuclearNF-κB p65 in the ipsilateral hemisphere of untreated injured mice. Densitometric analysis showed increased nuclear p65 levels at 1, 3, 6 and 24 h post-injury, with a peak at around 3 h. ^#^ *P*<0.05 versus 3 h (n = 5 mice/time point). (**E**) Representative immunoblots and densitometric analysis showed a significantly greater decrease in nuclear NF-κB p65 levels in the ipsilateral hemispheres of the wogonin-treated injured mice than in the vehicle-treated injured mice at day 1. (**F**) Representative gel shift analysis showing NF-κB DNA-binding activity from a sham control mouse (*lane 3*), vehicle-treated injured mouse (*lane 4*), and wogonin-treated injured mouse (*lane 5*) at day 1. Competition assays for NF-κB DNA-binding activity were performed with a 50-fold excess of unlabeled competitor NF-κB consensus oligonucleotides (*lane 1*). For the supershift assay, an antibody targeting the p65 subunit of NF-κB was incubated with a nuclear protein sample before the binding reaction (*lane 2*). Quantification analysis showed that wogonin treatment induced a significantly greater decrease in NF-κB binding activity, expressed in arbitrary densitometric units (ADU), than that induced by vehicle treatment, measured by EMSA.Values are reported as means ± SEM; *P<0.05, **P<0.01, ***P<0.001 versus sham controls, and ^†^ *P*<0.05, ^†††^ *P*<0.001 for wogonin-treated versus vehicle-treated TBI mice (n = 7 mice/group).

We next assessed the effects of wogonin treatment on the expression of inflammatory mediators. Since our previous studies showed that the mRNA and protein expression of most cytokines peaked at 6 and 24 h after TBI, respectively [24], we chose to evaluate the wogonin effect on mRNA and protein expression of various inflammatory mediators at 6 h and 1 day post-injury. Protein expression of interleukin (IL-1β), IL-6, macrophage inflammatory protein (MIP)-2 and monocyte chemoattractant protein (MCP)-1 was detected using enzyme-linked immunosorbent assay (ELISA) whereas that of COX-2 was detected using western blot. mRNA expression of all the inflammatory mediators was detected by real-time quantitative reverse transcription polymerase chain reaction (RT-PCR) analysis. Basal levels of IL-1β, IL-6, MIP-2, MCP-1, and COX-2 were low in sham control brains. However, TBI induced a significant increase in IL-1β, IL-6, MIP-2, MCP-1, and COX-2 protein expression in the injured cortex of both vehicle-treated and wogonin-treated mice compared with the sham control at day 1 post-injury (Figs. 5A–D & 6A). Injured cortices from wogonin-treated mice exhibited significantly reduced IL-1β, IL-6, MIP-2, and COX-2 protein levels compared with the vehicle group (IL-1β: 49.1±12.7 versus 84.9±9.4 pg·mg^−1^ protein, *P*<0.05, Fig. 5A; IL-6: 86.5±8.1 versus 130.7±11.6 pg·mg^−1^ protein, *P*<0.01, Fig. 5B; MIP-2: 156.5±17.4 versus 221.7±11.3 pg·mg^−1^ protein, *P*<0.01, Fig. 5C; COX-2: 70.6% of the vehicle group, *P*<0.05, Fig. 6A). Though MCP-1 protein levels appeared to follow the same trend, the changes were not statistically significant (Fig. 5D). Similarly, mRNA levels of IL-1β, IL-6, MIP-2, MCP-1 and COX-2 increased significantly in the injured cortices of vehicle-treated mice at 6 h post-injury compared with sham controls (*P*<0.05 for all values; Figs. 5E–H & 6A). The increase in IL-1β, IL-6, MIP-2, and COX-2 mRNA levels was significantly attenuated by wogonin treatment. IL-1β, IL-6, MIP-2, and COX-2 mRNA levels in the wogonin-treated injured cortex were 56.0% (*P*<0.01; Fig. 5E), 73.5% (*P*<0.05; Fig. 5F), 54.2% (*P*<0.01; Fig. 5G), and 47.7% (*P*<0.01; Fig. 6B) of the vehicle group at 6 h, respectively. In contrast, no significant differences were found for MCP-1 mRNA (Fig. 5H).

### Post-injury wogonin administration reduces MMP-9 enzymatic activity after TBI

MMP-9 activity was significantly increased in both vehicle-treated and wogonin-treated injured brains at day 1 post-TBI (Fig. 6C). MMP-9 activity was significantly decreased in wogonin-treated mice compared with vehicle-treated mice (*P*<0.05).

### Post-injury wogonin administration reduces NF-κB binding activity after TBI

NF-κB is a major transcription factor that regulates the expression of several genes involved in the inflammatory response. To investigate the time-course expression of NF-κB activation, western blot analyses were performed with nuclear extracts. Basal nuclear NF-κB p65 levels were low in the cortex of the sham-injured animals. Activation of NF-κB, as indicated by the nuclear translocation of p65 was observed at 1, 3, 6 and 24 h after injury, with the peak level being observed at 3 h (*P*<0.05 for all values) (Fig. 6D). Wogonin treatment significantly reduced the injury-induced increased nuclear levels of NF-κB to 55.3% (*P*<0.05) of vehicle-treated brains at day 1 post-TBI (Fig. 6E). We further used electrophoretic mobility shift assays (EMSAs) to examine whether wogonin affected the DNA binding activity of NF-κB. NF-κB was present at low levels in sham control brains. Compared with the sham group, NF-κB binding activity was significantly increased in the vehicle-treated TBI group at day 1 post-TBI (Fig. 6F). Wogonin treatment significantly reduced NF-κB binding activity. NF-κB binding activity in the injured brains of wogonin-treated mice was 61.3% (*P*<0.05) of vehicle-treated control brains at day 1.

### Post-injury wogonin administration reduces TLR-4 protein expression after TBI

**Figure 7:**
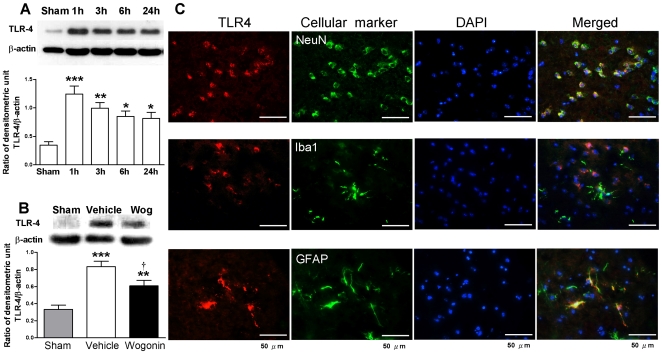
Effects of 40 mg·kg^−1^ wogonin on TLR4 expresssion. (**A**) Representative immunoblots of toll-like receptor (TLR)-4 protein in the ipsilateral hemisphere from untreated injured mice. Bar graphs of densitometry analysis of the protein bands showed increased TLR4 levels at 1, 3, 6 and 24 h post-injury (n = 5 mice/time point). (**B**) Representative immunoblots showing TLR-4 protein in the ipsilateral hemisphere from a sham-injured control, a wogonin-treated injured mouse, and a vehicle-treated injured mouse at day 1 post-TBI. Bar graphs of densitometry analysis of protein bands showed a significant decrease in TLR4 protein levels in the ipsilateral hemispheres of wogonin-treated mice at day 1 post-TBI compared with vehicle-treated mice. (**C**) Identification of TLR4- positive cells 1 day post-injury in the peri-contusion margin by immunofluorescence labeling. TLR-4 immunoreactivity is shown in red, and immunolabeling of NeuN (a cell marker for neurons), anti-ionized calcium binding adaptor molecule 1 (Iba1, a cell marker for microglia), or GFAP (a cell marker for astrocytes) is shown in green. Yellow labeling indicates co-localization. TLR4 was co-localized in neurons and astrocytes, and poor colocolization was observed in microglia. Sections were stained with DAPI (blue) to show all nuclei. The scale bar is 50 µm. Values are reported as means ± SEM; *P<0.05, **P<0.01, ***P<0.001 versus sham controls, and ^†^ *P*<0.05 for wogonin-treated versus vehicle-treated TBI mice (n = 7 mice/group).

Since activation of TLR4 stimulates the activation of NF-κB, we next examined the effects of wogonin on TLR4 protein expression. Basal TLR4 expression was low in sham control brains. The expression of TLR4 significantly increased starting from 1 h after injury (*P*<0.05), and remained high between 3 h to 1 day post-TBI (*P*<0.05 for all values) (Fig. 7A). At day 1 post-injury, TLR4 protein expression was significantly increased in the injured cortex of both vehicle-treated and wogonin-treated mice compared with sham controls (Fig. 7B). Dual-label immunofluorescence demonstrated that TLR4 was co-localized in neurons and astrocytes, and poor co-localization was observed in microglia, in the peri-contusional area (Fig. 7C). Wogonin treatment significantly reduced TLR4 levels to 73.0% (*P*<0.05) of vehicle-treated control brains (Fig. 7B).

## Discussion

This study shows for the first time that wogonin administered 10 min after CCI reduces neuronal damage and cerebral edema and improves long-term sensory-motor recovery after TBI in mice. These beneficial effects are associated with a reduction in mRNA transcripts and protein expression of TLR4/NF-κB pathway-related mediators. Our results demonstrate that wogonin treatment is effective at attenuating the severity of TBI in mice, both clinically and neuropathologically.

TBI induces neurological deficits and can result in functional impairment as well as dependence. In clinical studies, the functional outcome following a traumatic insult is one of the most important parameters. We observed that wogonin treatment caused a significant reduction in both sensory-motor deficits (assessed by mNSS) and motor dysfunction (assessed by rotarod and beam walk tests) up to 4 weeks post-injury, indicating that wogonin was highly effective in providing long-lasting protection to the traumatized brain. We utilized rotarod and beam walk tests to measure motor coordination and balance [24], [25], and mNSS to evaluate overall neurological deficits [24]. Notably, we found that a single dose of wogonin reduced functional deficits caused by experimental TBI in mice as early as 1 day post-injury and promoted sustained improvement of motor-sensory functions over a 4-week post-injury period. The protection afforded by wogonin was associated with a decrease in neuronal damage and apoptotic cell death at day 1, and a reduction in contusion volume at both day 1 and day 28 in the cortical regions involved in motor and sensory functions. These findings suggest that wogonin effectively attenuated the pathological events leading to post-traumatic deficits during the first 24 h, which consequently led to a better prolonged recovery of neurologic function.

TLR4-mediated NF-κB signaling plays a vital role in the initiation of cerebral inflammation in several central nervous system (CNS) diseases, such as inflammatory or autoimmune CNS diseases, and cerebral ischemic injury [2], [26]. The activation of NF-κB leads to transcription of many pro-inflammatory genes that encode cytokines, chemokines, and enzymes such as COX-2 and MMP-9, mediators that are involved in the development of secondary brain injury following TBI [27], [28]. The upregulation of cytokines and chemokines, e.g., IL-1β, IL-6, and MIP-2, may activate microglia, initiate the infiltration of inflammatory cells into the brain, and trigger a series of events ultimately leading to neuronal death [1], [29], [30]. In addition, MMP-9 can degrade the neurovascular matrix, leading to edema and tissue injury [28], [31]. Induction of COX-2 up-regulates prostaglandin production, generates free radical species, and contributes to edema and neuronal death [32]. Here, we showed that wogonin treatment could attenuate TBI-induced reduced TLR4/NF-κB signaling pathway activation in the injured brain. It is possible that wogonin suppressed microglia activation and neutrophil infiltration and reduced injury-induced IL-1β, IL-6, MIP-2, MMP-9, and COX-2 expression, thereby ameliorating brain damage. Our results are in agreement with previous studies demonstrating that suppression of TLR4/NF-κB signaling by anti-inflammatory agents, including statins and progesterone, reduced TBI-induced brain damage [4], [5]. There is abundant evidence showing that wogonin attenuates several inflammatory processes known to be important during TBI. For example, wogonin has been shown to alleviate inflammatory processes by decreasing prostaglandin E2 production and COX-2 expression in macrophages [10]. Wogonin inhibited MCP-1 gene expression in human endothelial cells and suppressed MMP-9 expression in human aortic smooth muscle cells [33], [34]. It also ameliorated myocardial ischemia/reperfusion injury, suppressed activation of NF-κB and p38 mitogen-activated protein kinase and inhibited MCP-1 expression *in vivo* [35]. In the CNS, wogonin attenuated LPS-induced production of NO and pro-inflammatory cytokines in cultured microglia and astrocytes [15], [17], [36] and suppressed microglial cell migration via inhibition of NF-κB activity [13]. Additionally, it exhibited inhibitory effects on inducible nitric oxide synthase (*iNOS*) protein expression and MMP-9 enzyme activity in glioma cells [37]. These properties of wogonin may contribute to its neuroprotective effects. Indeed, i*nhibition* of *microglia by wogonin reduced cytotoxicity* when *cocultured* with *PC12* cells [11]. *In vivo* experiments also showed that wogonin treatment protected against brain damage by reducing the production of inflammatory mediators, e.g., tumor necrosis factor (TNF)-α and *iNOS*, preventing the death of hippocampal neurons in cerebral ischemia [11], and inhibiting the activation of microglia in excitotoxic brain injury [11].

We observed significant differences in brain water content and EB extravasation between wogonin-treated and vehicle-treated mice, providing evidence for the effects of wogonin on BBB permeability and brain edema after TBI. Global cerebral edema, or BBB dysfunction, has been reported as a major risk factor for poor outcomes after TBI [38]. BBB dysfunction may potentially allow circulating cells and many blood-borne substances into the brain, thus augmenting cerebral inflammatory responses and leading to further neuronal damage and edema formation. Accumulating evidence shows that inflammation and activation of MMPs play key roles in the disruption of the BBB and brain edema formation after injury [28], [38]–[40]. Furthermore, multiple studies have shown that suppression of inflammation inhibited BBB disruption and edema [5], [41], [42]. MMP-9 functions to degrade the extracellular matrix, including major components of the basal lamina and tight junctions as well as interendothelial tight junction proteins, causing BBB disruption after TBI [28], [38]. In addition, excessive accumulation of leukocytes causes the *release of cytotoxic enzymes*, inflammatory mediators, and *reactive oxygen species, thereby* potentially damaging the microvascular endothelium and leading to BBB disruption and edema [31], [38], [39]. In the current study, we showed that wogonin treatment decreased the number of microglia, macrophages, and neutrophils recruited to the injured areas of the brain, reduced NF-κB activation and translocation to the nucleus, and interacted NF-κB binding activity, expression of inflammatory mediators (IL-1β, IL-6, MIP-2, and COX-2), and MMP-9 activity in the injured brain. This reduction in inflammation was associated with the protection of 2 tight junction proteins, ZO-1 and claudin-5, suggesting that wogonin may act to protect endothelial tight junctions, thereby keeping the BBB intact. Hence, the anti-edematous effect of wogonin observed in our study is likely to be related to the inhibition of inflammation. However, the beneficial effects of wogonin may be in part due to the prevention of free radical and oxidant formation since wogonin exerts potent antioxidant effects both *in vitro* [15], [18] and *in vivo* [43], [44]. Further investigations are needed to clarify the mechanisms underlying the anti-edematous effects of wogonin.

We found that a single post-injury injection of 40 mg·kg^−1^ wogonin protected against brain injury but did not induce renal or liver toxicity in mice. The neuroprotective potential of wogonin has been demonstrated in animal models of global and focal ischemia as well as excitotoxic injury by systemic kainate injection in rats. The effective doses ranged from 1 dose of 10 mg·kg^−1^ (10 mg·kg^−1^ given 10 min after induction of global ischemia) to 2 doses of 20 mg·kg^−1^ (20 mg·kg^−1^ given 30 min before and 4 h after induction of focal ischemia) [16], [17], [33]. Nevertheless, all previous studies only tested the efficacy of wogonin at fairly early time points (1 to 7 days) after treatment. In contrast, our study investigated structural parameters (infarct size), functional outcomes, and physiological parameters (body weight, renal function, and liver function) over a period of up to 1 month. Furthermore, we found that post-injury wogonin treatment reduced cerebral edema, BBB permeability, and apoptotic cell death, which are major events that occur in the secondary injury phase of TBI. Wogonin has a good safety profile, and it is already used in humans in combination with other flavonoids extracted from *S. baicalensis* Georgi for several different indications [9], [45], [46]. These properties may facilitate clinical applications of wogonin to human TBI patients. Further investigations will be required to establish the optimal time window for treatment, route of administration, and therapeutic efficacy for different injury magnitudes and models.

In conclusion, our findings indicate that post-injury treatment with wogonin leads to improved long-term functional and histological outcomes and reduced brain edema in a clinically relevant model of TBI. This improvement was associated with attenuated expression of TLR4/NF-κB-pathway related mediators, including upstream factors (TLR4/NF-κB) and downstream factors (IL-1β, IL-6, MIP-2, and COX-2 expression, and MMP-9 activity), suggesting that the neuroprotective effects of wogonin following TBI may be mediated, in part, through modulation of the TLR4/NF-κB signaling pathway. Wogonin treatment may prove to be advantageous because chronic dosing is not required, wogonin has low toxicity, and it can be easily administered in emergency situations. Thus, wogonin could be a potential therapeutic in the treatment of TBI.

## Materials and Methods

### Surgical procedures

All animal procedures were approved by the Animal Research Committee at Cheng Hsin General Hospital (Animal permit number CHGH-98-003), and all procedures conformed to the Guide for the Care and Use of Laboratory Animals published by the US National Institutes of Health (NIH Publication No. 85–23, revised 1996). Animals were housed in groups in a temperature (21–25°C)- and humidity (45–50%)-controlled room with a 12-h light/dark cycle and *ad libitum* access to pellet chow and water. A previously described CCI injury model was utilized [47]. Eight-week-old male C57BL/6 mice (22–25 g body weight) were intraperitoneally anesthetized with sodium pentobarbital (65 mg·kg^−1^; Rhone Merieux, Harlow, UK) and placed in a stereotaxic frame. A 5-mm craniotomy was performed over the left parietal cortex, centered on the coronal suture and 3 mm lateral to the sagittal suture. Considerable care was taken to avoid injury to the underlying dura. Injury was induced using a pneumatic piston with a rounded metal tip (2.5 mm diameter) which was vertically angled (22.5°) so that the tip was positioned perpendicular, with the brain surface at the center of the craniotomy. A velocity of 4 m·s^−1^ and a deformation depth of 2 mm below the dura were used. The bone flap was immediately replaced and sealed, and the scalp was sutured closed. Body temperature was monitored throughout the surgery by using a rectal probe; temperature was maintained at 37.0±0.5°C using a heated pad. Mice were placed in a heated cage to maintain body temperature while recovering from anesthesia.

Sham-operated mice received craniotomy as described before, but without CCI; the impact tip was placed lightly on the dura before sealing the wound. After the trauma or sham surgery, animals were housed under the conditions mentioned above.

### Experimental protocol and dose selection

All animals were randomized into 1 of 3 groups (sham injury, CCI+vehicle, CCI+40 mg·kg^−1^ wogonin). Surgery/injury, behavioral testing, and tissue analyses were all performed by different individuals. Behavioral testing and tissue analyses were performed by individuals blinded to the treatment group. Wogonin (Wako Pure Chemical Industries, Osaka, Japan) dissolved in 30% dimethyl sulfoxide (DMSO, 0.1 mL) or a corresponding volume of vehicle (30% DMSO) was administered intraperitoneally 10 min following injury. Testing after injury was completed as follows: 1) behavioral testing at days 1, 4, 7, 14, 21, and 28 (n = 8 mice/group); 2) cresyl violet staining at days 1 and 28 (n = 8 mice/group); 3) histology, EB quantification, determination of brain water content, western blot analysis, ELISA and EMSAs at day 1 (n = 7 mice/group); and 4) real–time quantitative RT-PCR at 6 h (n = 7 mice/group). To determine the optimal dose of wogonin, a pilot study was performed using 3 different doses (20, 40, and 50 mg·kg^−1^) administered 10 min after injury, and neurological deficits were evaluated as main outcomes. The results showed a significant effect of wogonin at 40 mg·kg^−1^, with no further increase at 50 mg·kg^−1^. Therefore, on the basis of these data, a dose of 40 mg·kg^−1^ was chosen for all subsequent experiments.

The temporal profile of TLR4 expression and NF-κB activation was evaluated by western blot in another group of injured and sham-injured mice. The purpose of this analysis was to determine whether increased TLR4 expression and NF-κB activation precede increased mRNA of pro-inflammatory mediators. Following CCI operation, western blot using whole-tissue or nuclear extracts was performed at 1, 3, 6 and 24 h (n = 5 for both TLR4 and NF-κB expression at each time point). Ten additional sham-operated rats were used for the controls (n = 5 for both TLR4 and NF-κB western blot).

### Neurological functional evaluation

Behavioral testing was performed before CCI and at 1, 4, 7, 14, 21, and 28 days after CCI. The battery of tests consisted of the rotarod motor test, mNSS assessment, and beam walk test. Animals were pre-trained for 3 days for both the rotarod and beam walk tests.

#### Rotarod test

An accelerating rotarod was used to measure motor function and balance in mice [24]. Each mouse was placed on the rotarod cylinder, and the time that the animal remained on the rotarod was measured. Speed was slowly increased from 4 rpm to 20 rpm within 5 min. A trial would be ended if the animal fell off the rungs or gripped the device and spun around for 2 consecutive revolutions without attempting to walk on the rungs. One hour before CCI, the mean duration on the device was recorded with 3 rotarod measurements as pre-injury baseline values. Post-injury latencies were expressed as percentages of their respective baseline values to reduce interanimal variability.

#### Modified neurological severity score

The mNSS is a composite of motor, sensory, reflex, and balance tests [24]. One point was scored for the inability to perform each test or for the lack of a tested reflex; thus, the higher the score, the more severe the injury. Neurological function was graded on a scale of 0–18 (normal score, 0; maximal deficit score, 18).

#### Beam walk test

The beam walk test was utilized to evaluate fine motor coordination and function [24]. Mice escaped a bright light and loud white noise by walking along an elevated (50 cm) narrow wooden beam (0.8 cm×100 cm) to enter a darkened goal box at the opposite end of the beam. The time required for the mouse to reach the goal box (not to exceed 60 s) and hindlimb performance as it traversed the beam (based on a 1 to 7 rating scale) were recorded. A score of 7 was given when animals traversed the beam with 2 or less foot slips; 6 was given when animals traversed the beam with less than 50% foot slips; 5 was given for more than 50% but less than 100% foot slips; 4 was given for 100% foot slips; 3 was given for traversal with the affected limb extended and not reaching the surface of the beam; 2 was given when the animal was able to balance on the beam but not traverse it; 1 was given when the animal could not balance on the beam. Three trials were recorded 1 h before CCI (baseline) and each day after CCI. The mean values of latency and score for each day were computed.

### Evaluation of metabolic characteristics

Following terminal anesthesia, venous blood was collected via direct right atrial puncture. The obtained blood was centrifuged (3500 rpm for 5 min), and the serum was stored at −20°C until analysis. A chemistry analyzer (*Synchron* Clinical System LX20; *Beckman Coulter*, Fullerton, *CA)* was used to measure serum BUN and CRE to assess renal function, and AST and ALT to assess liver function.

### Tissue processing and histology

Following terminal anesthesia, mice were processed for histology by transcardial perfusion with phosphate-buffered saline (PBS) followed by 4% paraformaldehyde. All solutions were maintained at pH 7.4 and 4°C. Brains were removed, post-fixed in 4% paraformaldehyde overnight, and then transferred to PBS containing 30% sucrose and 0.1% sodium azide (Sigma-Aldrich, St. Louis, MO) for cryoprotection. Coronal sections were cut in a cryostat at 10 µm from the level of the olfactory bulbs to the visual cortex and used for cresyl violet histology, FJB staining, TUNEL staining, or immunohistochemistry.

### Contusion volume and hemispheric enlargement analysis

Contusion volumes and hemispheric enlargement were quantified using coronal sections stained with cresyl violet at 20 rostral-caudal levels that were spaced 200 µm apart. Sections were digitized and analyzed using a 1.5× objective and Image J software (Image J, National Institutes of Health, Bethesda, MD). The contusion area was calculated using all cresyl violet-stained sections containing contused brain as previously described [24], and the volume measurement was computed by summation of the areas multiplied by the interslice distance (200 µm). Brain edema was measured by determining the percentage of hemispheric enlargement, which was calculated using the following formula: [(ipsilateral hemisphere volume−contralateral hemisphere volume)/contralateral hemisphere volume]×100 [48]. Analysis was conducted by two experimenters who were blinded to all animal characteristics. Inter-rater reliability in contusion volumes and hemispheric enlargement was well within 10%.

### Evaluation of blood-brain barrier permeability

One day after TBI, BBB permeability was evaluated by measuring EB extravasation. Briefly, EB dye (4 mL·kg^−1^ in 2% saline) was administered via the tail vein and allowed to circulate for 60 min. To wash out intravascular EB, the animals were then perfused with saline through the left ventricle at a pressure of 110 mmHg until colorless fluid was obtained from the right atrium. Brains were removed, and ipsilateral hemispheres were cut into 4-mm-thick sections (2 mm from the frontal pole) and weighed. For the extraction of EB from brain tissues, hemispheres were placed in 1 mL of 60% (w/v) trichloroacetic acid and homogenized by sonication. Homogenates were centrifuged at 4500 rpm for 15 min, and the supernatants were diluted with ethanol (1∶4). The absorbance of each supernatant for the EB dye was measured at 620 nm using a spectrophotometer. EB concentrations were calculated and expressed as µg·g^−1^ brain tissue against a standard curve.

### Brain water content

Brain water content represents brain edema, which forms as a consequence of BBB breakdown and post-injury inflammation. Mice were re-anesthetized and decapitated at day 1, a time point associated with maximal edema formation following experimental TBI [21]. Brain water content was measured by the tissue-drying method in a 4-mm coronal tissue section of the ipsilateral hemisphere, 2 mm from the frontal pole. The water content of a sample was determined by measuring the difference between wet weight (fresh tissue weight) and dry weight and expressed as a fraction of the wet weight [42], [48]. Brain samples were immediately weighed on an electric analytical balance to obtain the wet weight and then dried at 100°C for 24 h to obtain the dry weight. The water content of each sample (% water content) was calculated using the following formula: (wet weight−dry weight)/wet weight×100%.

### FJB histochemistry

FJB is a polyanionic fluorescein derivative that binds with high sensitivity and specificity to degenerating neurons, and staining was done as previously described [49], with some modifications. Briefly, sections were first incubated in a solution of 1% NaOH in 80% ethanol for 5 min and then rehydrated in graded ethanol (75, 50, and 25%; 5 min each) and distilled water. Sections were then incubated in 0.06% KMnO4 for 10 min, rinsed in distilled water for 2 min, and incubated in a 0.0004% solution of FJB (Chemicon, Temecula, CA) for 30 min. Sections were observed and photographed under a fluorescence microscope (Olympus BX-51; Olympus, Tokyo, Japan) with blue (450–490 nm) excitation light.

### TUNEL staining

TUNEL assay was performed using a commercial kit that labels DNA strand breaks with fluorescein isothiocyanate (FITC; In Situ Cell Death Detection Kit, Roche Molecular Biochemicals, Mannheim, Germany). Sections were pretreated with 20 µg/mL proteinase-K in 10 mM Tris-HCl at 37°C for 15 min. The slices were then washed in distilled water and PBS and incubated in 0.3% hydrogen peroxide solution. Each section was incubated with 50 µL of TUNEL reaction mixture with terminal deoxynucleotidyl transferase (TdT) for 60 min at 37°C under humidified conditions. Sections were observed and photographed under a fluorescence microscope with blue (450–490 nm) excitation light. Negative controls were obtained by omitting the TdT enzyme.

### Immunohistochemistry

All sections were dried, rehydrated in PBS, fixed in 4% paraformaldehyde for 20 min, and rinsed in PBS. Sections were then quenched in a solution of 10% methanol/10% hydrogen peroxide in distilled water for 5 min before washing 3 times in Tris-buffered saline (TBS; Sigma). Sections were blocked for 60 min in TBS containing 0.2% Triton X-100 (TXTBS; Sigma) and 3% normal goat serum (NGS; Dako, Carpinteria, CA) and then incubated overnight at 4°C with the respective primary antibody (rabbit polyclonal anti-myeloperoxidase [MPO], a neutrophil marker [Dako], or rabbit anti-Iba1, a microglia marker [Wako]) in TXTBS containing 1% NGS. After 3 washes in TBS, sections were incubated with a biotinylated secondary antibody (biotinylated anti-rabbit IgG; Vector, Burlingame, VT) at a 1∶200 dilution in TBS containing 1% NGS for 3 h, followed by 3 washes in TBS. Detection of the primary antibody was facilitated by use of a streptavidin-biotinylated horseradish peroxidase complex kit (Dako), in which samples were incubated with diaminobenzidine in TBS containing 1% NGS for 2 h, followed by 3 washes in TBS and 2 washes in Tris non-saline (TNS). Sections were developed with diaminobenzidine in TNS containing 0.03% hydrogen peroxide, and excess stain was removed by washing in TNS 3 times. The specificity of the staining reaction was assessed by several control procedures, including omission of the primary antibody and substitution of the primary antibody with non-immune rabbit serum.

In order to assess the cellular source of TLR4 after TBI, double immunofluorescence labeling was performed by simultaneous incubation of rabbit or goat polyclonal anti-TLR4 (1∶100 dilution; Santa Cruz Biotechnology, Santa Cruz, CA) with mouse anti-neuronal nuclei antigen (1∶100 dilution; NeuN, a neuronal marker; Chemicon, Temecula, CA), rabbit polyclonal anti-Iba1 (1∶100 dilution; a microglia marker; Wako), or rat monoclonal anti-glial fibrillary acidic protein (GFAP, 1∶400 dilution; an astrocyte marker; *Zymed Laboratories*, South San Francisco, CA) overnight at 4°C. Sections were then washed, incubated with Alexa Fluor 488 and Alexa Fluor 594 (1∶400; Molecular Probes, Eugene, OR) for 2 h, and examined under a fluorescence microscope (Olympus BX-51; Olympus, Tokyo, Japan).

### Quantification of FJB, TUNEL, MPO, and Iba-1 staining

FJB, TUNEL, MPO, and Iba-1 staining was quantified on stained sections at the level of 0.74 mm from the bregma. Three sections per animal were viewed and photographed under a microscope. FJB-, TUNEL-, MPO-, and Iba1-positive cells were counted by sampling an area of 1280×1024 µm^2^ (for FJB, MPO, and Iba1 staining) or 1600×1200 µm^2^ (for TUNEL staining) immediately adjacent to the cortical contusion margin in 3 randomly selected, non-overlapping fields using a magnification of ×200. The total number of FJB-, MPO-, and Iba1-positive cells was expressed as the mean number per field of view. Quantification of TUNEL staining was expressed as the percentage of nuclei that were stained by the TUNEL method divided by the total number of DAPI-stained nuclei. Analysis was conducted by 2 independent experimenters who were blinded to all animal characteristics. Interrater reliability in cell counts was within 10%.

### Western blot analysis

Mice were re-anesthetized and decapitated 1 day after CCI or sham operation for western blot analysis. A 4-mm coronal section was taken from the injured area over the parietal cortex, and then homogenized in ice-cold protein extraction reagents (T-PER reagent; Pierce Biotechnology, Rockford, IL) containing a complete mini protease inhibitor cocktail (Roche). Nuclear extracts were prepared using a nuclear protein extraction reagent kit (Marligen Biosciences Inc., Rockville, MD). Western blots were performed as previously described [48]. Briefly, equal amounts of protein were separated by sodium dodecyl sulfate-polyacrylamide gel, transferred to Immobilon-P membranes (Millipore, Billerica, MA), blocked using 5% milk in PBS containing 0.1% Tween-20, and probed with primary antibodies at 4°C overnight. Afterwards, the membranes were washed and incubated with horseradish peroxidase-linked anti-rabbit or anti-mouse secondary antibodies (1∶1000 dilution; Santa Cruz Biotechnology) for 2 h. The relative intensity of each protein signal was normalized to the corresponding β-actin intensity and quantified by densitometry analysis using Image J software. The following primary antibodies and dilutions were used: 1) rabbit polyclonal anti-claudin-5 (1∶1000 dilution; Invitrogen, Camarillo, CA); 2) rabbit polyclonal anti-ZO-1 (1∶200 dilution; Invitrogen); 3) rabbit polyclonal anti-COX-2 (1∶1000 dilution; Cayman Chemical, Ann Arbor, MI); 4) rabbit polyclonal anti-TLR4 (H-80; 1∶1000 dilution; Santa Cruz Biotechnology); 5) rabbit polycloncal anti NF-κB p65 (1∶1000 dilution, Santa Cruz Biotechnology); 6) mouse monoclonal anti-β-actin (1∶5000 dilution; Sigma); 7) rabbit polycloncal anti-Lamin A/C (1∶1000 dilution, Santa Cruz Biotechnology).

### Enzyme-linked immunosorbent assay

Brains from injured or sham control animals were removed without fixation after cervical dislocation 1 day following surgery. A 4-mm coronal section was taken from the injured area over the parietal cortex, snap-frozen in liquid nitrogen, and stored at −70°C until use. ELISAs were performed to detect IL-1β, IL-6, MIP-2, and MCP-1 in brain homogenates using commercially available kits (R&D Systems, Minneapolis, MN). Tissue homogenates were diluted to correspond with the linear portion of the respective standard curves as determined in preliminary studies. All samples and standards were assayed in duplicate according to the manufacturer's instructions. Tissue cytokine and chemokine concentrations were expressed as pg antigen per mg protein.

### Gelatin zymography

One day after induction of TBI, mice were euthanized by cervical dislocation and brains were removed without fixation. Zymography was performed as previously described [48]. Briefly, equal amounts of protein (prepared in the same manner as the samples for western blot analysis) were separated on a 10% Tris-glycine gel copolymerized with 0.1% gelatin as substrate. After separation, the gel was washed twice in distilled water (30 min each wash) and then, proteins within the gel were renatured by incubation with 2.5% Triton-X-100 buffer for 1 h at room temperature. After incubating with developing buffer (0.05 M Tris-HCl pH 7.5, 0.2 M NaCl, 5 mM CaCl_2_, 0.05% Brij-35, and 0.2 mM NaN_3_) at 37°C for 24 h, the gel was stained with 0.05% Coomassie R-250 dye (Sigma) for 30 min followed by destaining. Gelatinolytic activity (MMP-9: ∼97 kDa) was indicated by the detection of clear bands at the appropriate molecular weight.

### Real-time quantitative RT-PCR

Six hours after injury or sham surgery, mice were euthanized by cervical dislocation and brains from both injured and sham animals were removed without fixation. A 4-mm coronal section was taken from the injured area over the parietal cortex, snap-frozen in liquid nitrogen, and stored at −70°C until use. Total RNA was extracted from brain tissues using the RNeasy Mini Kit (Qiagen, Valencia, CA) and subsequently subjected to reverse transcription using SuperScript II RNase H reverse transcriptase (Invitrogen). Real-time quantitative RT-PCR analysis was performed on an ABI PRISM 7900 sequence detector (Applied Biosystems, Foster City, CA). Primers and probes for IL-1β (TaqMan Gene Expression Assay ID Mm00434228_ml), IL-6 (Mm00446190_ml), MIP-2 (Mm00436450_m1), MCP-1 (Mm00441242_m1), and COX-2 (Mm00478374_m1) were purchased from Applied Biosystems. β-Actin (Rn00607939_s1) was used as endogenous control. Thermal cycling was initiated with a 2-min incubation at 50°C, followed by a 10-min denaturation step at 95°C and 40 cycles at 95°C for 15 s and 60°C for 1 min. Relative quantities of the candidate genes and β-actin rRNA were calculated using the previously described comparative threshold cycle (Ct) method [25].

### Electrophoretic mobility shift assay

Dissected cortices (prepared as in western blot analysis) were extracted using a nuclear protein extraction reagent kit. EMSAs were performed using a commercial kit according to the manufacturer's protocol (NF-κB EMSA kit; Panomics, Inc., Fremont, CA). Briefly, the double-stranded biotin-labeled NF-κB oligonucleotide probe (5′-AGTTGAGGGGACTTTCCCAGGC-3′) was incubated with the nuclear extracts in binding buffer and poly[d(I-C)] for 30 min on ice. Samples were separated by electrophoresis in 6% Tris-borate-EDTA (TBE) gels and transferred to nylon membranes. Oligonucleotides on the membranes were fixed for 3 min using a UV crosslinker. After incubating with blocking buffer at room temperature for 15 min, the membranes were reacted with streptavidin-conjugated horseradish peroxidase for another 15 min. After washing, the membranes were incubated with detection buffer at room temperature for 5 min, followed by incubation with chemiluminescent substrate solution for another 5 min. Shifted bands corresponding to the protein/DNA complexes were visualized relative to unbound double-stranded DNA after exposure to a radiographic film. The specificity of the binding reaction was evaluated by adding a 50-fold excess of unlabeled probe to the protein/DNA reaction mixture, which competes with the labeled DNA probe for binding to the protein. For the supershift assay, a primary antibody recognizing the p65 subunit of NF-κB (1∶1000 dilution, Santa Cruz) was incubated with the nuclear protein sample at 37°C for 1 h before performing the binding reaction. Optical densities of the bands were quantified using Image J software.

### Statistical analyses

Data are presented as the mean ± standard error of the mean (SEM). For comparisons among multiple groups, one-way or two-way analysis of variance (ANOVA), followed by post-hoc (Bonferroni) test, was used to determine significant differences. Differences between 2 groups were tested using the Student's *t*-test. Statistical significance was set at *P*<0.05.
