# Correction: Wogonin Improves Histological and Functional Outcomes, and Reduces Activation of TLR4/NF-κB Signaling after Experimental Traumatic Brain Injury

**DOI:** 10.1371/annotation/1f110857-27d7-4e83-9eb3-4e5f51950a26

**Published:** 2012-12-03

**Authors:** Chien-Cheng Chen, Tai-Ho Hung, Yen-Ho Wang, Chii-Wann Lin, Pei-Yi Wang, Chun-Yen Lee, Szu-Fu Chen

There were errors in the author affiliations. The affiliations should appear as shown here:

Chien-Cheng Chen^**1,2**^, Tai-Ho Hung^**3**^, Yen-Ho Wang^**4**^, Chii-Wann Lin^**1**^, Pei-Yi Wang^**2**^, Chun-Yen Lee^**2**^, Szu-Fu Chen^2,5*^

**1** Institute of Biomedical Engineering, National Taiwan University Taipei, Taiwan, Republic of China, **2** Department of Physical Medicine & Rehabilitation, Cheng Hsin General Hospital, Taipei, Taiwan, Republic of China, **3** Department of Obstetrics and Gynecology, Chang Gung Memorial Hospital at Taipei and College of Medicine, Chang Gung University, Taipei, Taiwan, Republic of China, **4** Department of Physical Medicine & Rehabilitation, National Taiwan University Hospital and National Taiwan University College of Medicine, Taipei, Taiwan, Republic of China, **5** Departments of Physiology and Biophysics, National Defense Medical Center, Taipei, Taiwan, Republic of China

